# Measuring quality in colorectal cancer surgery in low- and middle-income countries: The Clavien-Dindo classification in a Sri Lankan cohort

**DOI:** 10.1016/j.amsu.2022.104018

**Published:** 2022-06-18

**Authors:** S. Lindholm, S. Lindskogen, B. Gamage, G. Kurlberg, D. Ljungman

**Affiliations:** a Sahlgrenska Academy, University of Gothenburg, Gothenburg, Sweden; b Department of Surgery, Colombo South Teaching Hospital, University of Sri Jayewardenepura, Nugegoda, Sri Lanka; c Department of Surgery, Institute of Clinical Sciences, Sahlgrenska Academy, University of Gothenburg, Gothenburg, Sweden; d Region Västra Götaland, Department of Surgery, Sahlgrenska University Hospital, Gothenburg, Sweden

**Keywords:** Colorectal cancer, Sri Lanka, Clavien-Dindo classification, Postoperative complications, Surgical quality

## Abstract

**Background:**

The colorectal cancer (CRC) incidence is increasing in low- and middle-income countries (LMICs) as part of an ongoing epidemiological transition. Surgery is the main treatment and surgical services are scaled up to meet the need. This warrants the establishment of frugal systems to measure safety and quality of surgical care that are tailored for low-resource settings. The aim of this study was to test the applicability of the Clavien-Dindo classification (CDC) for measurement of surgical complications in an LMIC setting where medical records are paper-based.

**Material and methods:**

88 patients who underwent CRC resection at Colombo South Teaching Hospital, Sri Lanka, from January 2017 to January 2020 were included. Medical records were retrospectively reviewed for postoperative complications and the severity was graded using the CDC.

**Results:**

One or more postoperative complications (CDC ≥ grade II) occurred in 45.5% (n = 40) of the patients. The complications were distributed as grade II n = 46, grade III n = 3, grade IV n = 2 and grade V n = 0. The most common complication (22.7%, n = 20) was postoperative anemia treated with blood transfusion. The second most common complication was incisional surgical site infection (11.4%, n = 10).

**Conclusion:**

Postoperative outcome could be evaluated by using the CDC in a Sri Lankan facility based on retrospective review of medical records. This suggests that the CDC is a feasible standardized system appropriate for measuring surgical quality also in other LMICs. Identified fields for possible quality improvement at the study site were to limit blood transfusions and minimize treatment with antibiotics.

## Highlights

• Global health is putting focus on not only access to surgery, but also surgical quality and safety.
• The Clavien-Dindo Classification may be a standardized way of evaluating surgical quality in low-resource settings.
• The feasibility of Clavien-Dindo Classification in a retrospective cohort in Sri Lanka is demonstrated.

## Graphical abstract

**Figure:**
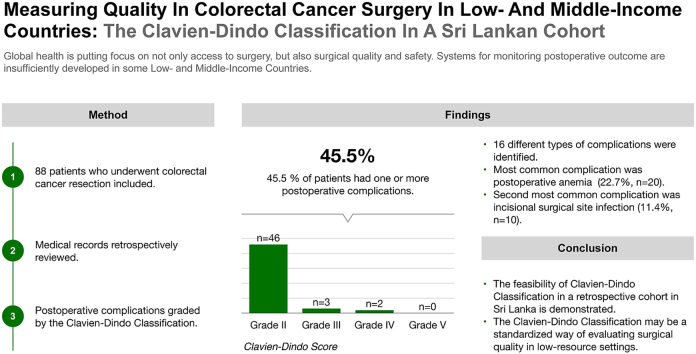

## 1 Introduction

Currently most Low- and Middle-Income Countries (LMICs) are experiencing a powerful epidemiologic transition from infectious diseases towards Non-Communicable Diseases (NCDs). One of the NCDs that is most strikingly representing the epidemiological transition is colorectal cancer (CRC) [1], globally, the third most common type of malignancy [2]. Surgery is the cornerstone of treatment for cure of CRC [3], as well as many other NCDs. Thus, the need for surgical care is rapidly increasing in LMICs. The Lancet Commission on Global Surgery (LCoGS) argued that surgical care needs to be prioritized, both regarding availability and safety [4]. Since the volume of surgical procedures in low-resource settings is increasing the surgical quality needs to be properly monitored. As many systems for measuring surgical quality are tailored to high income settings *Citron I. Et Al* recently presented a tool consisting of a number of quality indicators more approachable for low-resource settings [5].

As a supplement to crude postoperative mortality, frequently avertable postoperative complications should be included in measurement of outcome quality. Though, estimating impact of complications in a reproducible way in various settings can be problematic. The Clavien-Dindo Classification (CDC), a widely used validated scoring system for postoperative complications, is helpful in enabling such weighing of complications. In the CDC complications are classified as grade I–V, depending on the level of treatment required [6]. The CDC is one of the quality indicators suggested by *Citron I. Et Al* [5].

Sri Lanka is one of the LMICs undergoing the epidemiological transition in part manifested as a slowly increasing incidence of various malignancies, including CRC [7]. Registries on various malignancies exist in Sri Lanka, though these are insufficiently covering the surgical quality [8,9].

The aim of this study was to investigate short-term outcome after colorectal cancer surgery in a Sri Lankan facility using the Clavien-Dindo Classification to evaluate its applicability for measuring surgical quality also at medical institutions in LMICs where medical records are not fully developed. In addition we wanted to determine length of hospital stay (LOS) as an indicator of process quality [10]. To test the CDC results in practice we compare prevalence and severity of overall complications between patients <60 and ≥ 60 years of age, since age is a well-known risk factor for postoperative complications [11], taking in mind the relatively low median age (60 years) [12] of Sri Lankan CRC patients.

## 2 Material and methods

### 2.1 Study design

This is a retrospective single centre cohort study based on medical records of patients who have undergone resection for colorectal cancer (CRC) at Colombo South Teaching Hospital (CSTH) from the first of January 2017 to January 29, 2020. STROCSS guidelines were used in the reporting of the research [13].

### 2.2 Patient identification

In Sri Lanka patients are provided with a unique bed head ticket (BHT) number on each admission. All medical recording during the current hospitalization is marked with the BHT. At CSTH, all BHTs (medical records) are paper based and stored at the Medical Records Department for 12 years.

### 2.3 Study population

**Fig. 1:**
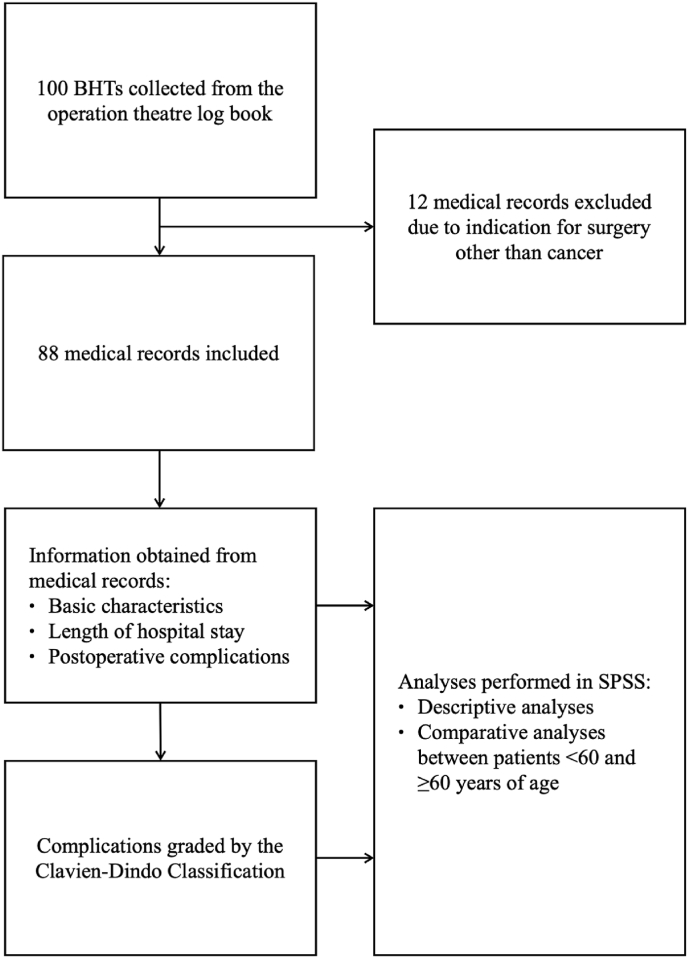
Flow chart of the method of the study. BHT = Bed head ticket; SPSS = Statistical Package for the Social Sciences.

100 BHTs, all consecutive colorectal resections, were collected from the surgical log book from February to March 2020 and submitted to the Medical Records Department for retrieval of medical records. Patients of both sex, all ages, all types of CRC surgeries, as well as both acute and elective surgeries were included. Patients excluded from the study were those with indication for surgery other than CRC (n = 12). Hence, 88 patients were finally included in the study (see Fig. 1).

### 2.4 Data collection

The study was registered at clinicaltrials.gov (https://clinicaltrials.gov/ct2/show/NCT05182762). Demographic, clinical and pathological characteristics of patients and details of surgeries and postoperative outcome were obtained from the medical records and documented in Excel. Length of hospital stay (LOS), defined as number of days following surgery to discharge, was calculated. The medical record was reviewed for postoperative complications and the treatment required. The severity of complications was graded according to the Clavien-Dindo Classification (CDC) [6] using a template from AssesSurgery [14]. Only complications of grade II or higher were included in this study as the documentation of grade I complications in medical records was considered too unreliable, with risk of description bias in line with many previous studies [15,16]. Time from surgery till discharge was used as a timeframe for postoperative complications, as defined by Lancet Commission on Global Surgery [4]. Urological complications that only resulted in catheter left at discharge did not have an obvious classification. Hence, such complications were classified as grade II, similarly to the Swedish Colorectal Cancer Registry [17]. Moreover, although incisional surgical site infections (SSI) are commonly classified as grade I, such infections were classified as grade II if systemic antibiotics were given, since pharmacological treatment belongs to grade II [6].

### 2.5 Statistical methods

Statistical Package for the Social Sciences (SPSS) version 26 was used for descriptive analyses of frequencies and to test differences in rate of overall complications and severe complications between patients <60 and ≥ 60 years of age, using the Chi-squared test or Fisher's exact test. P < 0.05 was considered statistically significant.

### 2.6 Ethical considerations

Ethical approval was provided by the ethics review committee of the institution (diary number: MO/PLEC/2020). Patients were anonymized using unique study identification numbers.

## 3 Results

### 3.1 Characteristics of patients and surgery

**Fig. 2:**
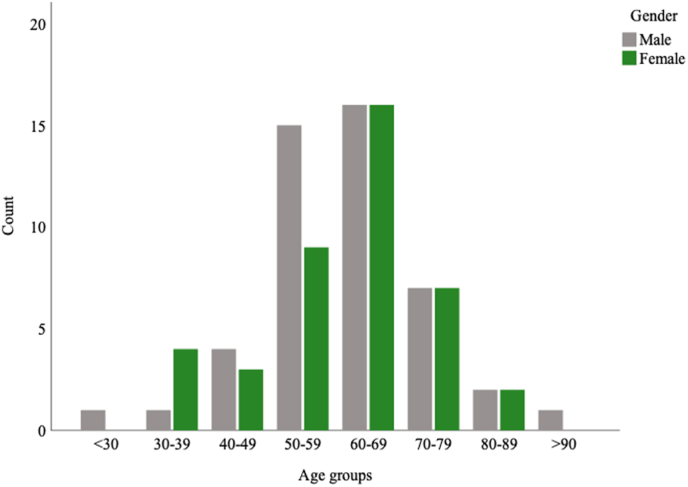
The study population divided into different age groups and gender.

53.4% (n = 47) of the patients were males and 46.6% (n = 41) were females. Mean age for males was 61.1 years and 60.4 for females. The age span was 23–92 years. The majority of patients (64%) were 50–69 years old. Age distribution is illustrated in Fig. 2. 40.9% (n = 36) of the patients were classified as ASA I, 54.5% (n = 48) as ASA II and 4.5% (n = 4) as ASA III. Table 1 summarizes characteristics of the surgeries.

**Table 1:** Characteristics of the surgeries performed.

		Number	%
**Tumour localization**	Colon	33	37.5
	Rectum	55	62.5
**Procedure**	Open	33	37.5
	Laparoscopic	49	55.7
	Converted	6	6.8
**Planning**	Acute	1	1.1
	Elective	87	98.9

### 3.2 Outcome

Altogether, one or more postoperative complications (≥ grade II) occurred in 45.5% (n = 40) of the patients. However, the number of complications was 51, as a patient can have more than one complication. The different complications were allocated as grade II n = 46, grade III n = 3, grade IV n = 2 and grade V n = 0 (Table 2). Since no patient developed grade IIIa or IVb complications, the grades are presented in the contracted forms, as grade III and IV. The most common complication, affecting 22.7% (n = 20) of the patients, was postoperative anemia treated with blood transfusion. At CSTH the indication for anemia correction postoperatively is generally Hb less than 9 g/dl (= 90 g/l). It was noticed during the data collection that most of these patients were anemic preoperatively, although the anemia became more severe after surgery. The second most common complication was incisional surgical site infection (SSI) (11.4%, n = 10), treated with antibiotics and sometimes also removal of clips at bedside. In four patients, some kind of urinary tract injury, accidental or inevitable due to tumor overgrowth, was documented in the operation note and they required prolonged use of urinary catheter (catheter at discharge). Four patients developed hypertension postoperatively, requiring antihypertensive medication. Three patients developed grade III complications; one case of wound rupture, another case of mechanical obstruction at stoma site and the third case was anastomotic insufficiency. The most severe complications occurred in two patients who developed respiratory dysfunction requiring ICU-management, and thus classified as grade IV. The first patient suffered from Acute Respiratory Distress Syndrome (ARDS) and was treated with Noninvasive Intermittent Positive Pressure Ventilation (NIPPV) and high amounts of oxygen. The second patient developed anastomotic insufficiency. In contrast to the other patient with anastomotic insufficiency, this patient required both reoperation (two times) and mechanical ventilation at ICU for eight days. There was no event of death among the patients.

**Table 2:** List of all postoperative complications (≥ grade II).

Clavien-Dindo grade	Type of complication	Treatment	Number	%
Grade II			46 (total)	52.3
	Anemia	Blood transfusion	20	22.7
	Incisional SSI	Antibiotics	10	11.4
	Urinary tract injury	Catheter at discharge	4	4.5
	Hypertension	Antihypertensive medication	4	4.5
	Confusion	Antipsychotic medication	1	1.1
	Hypotension	Noradrenaline infusion	1	1.1
	Atrial fibrillation	Antiarrythmic medication	1	1.1
	NSTEMI	Routine medication	1	1.1
	Infection of unknown source	Antibiotics	1	1.1
	ACLF	Various medicines*	1	1.1
	Urinary retention	α1-receptor antagonist	1	1.1
	Gastritis	Omeprazole	1	1.1
Grade III			3 (total)	3.4
	Wound rupture	Resuturing in G.A.	1	1.1
	Anastomotic insufficiency 1	Reoperation in G.A	1	1.1
	Obstruction at stoma sie	Reoperation in G.A	1	1.1
Grade IV			2 (total)	2.3
	ARDS	NIPPV at ICU	1	1.1
	Anastomotic insufficiency 2	Invasive ventilation at ICU	1	1.1
Grade V			0	0

* Lactulose, Vitamin K, Tranexamic acid, antibiotic prophylaxis and plasma products.

SSI= Surgical site infection, NSTEMI= Non ST elevation myocardial infarction, ACLF = Acute-on-chronic liver failure, ARDS = Acute respiratory distress syndrome, G.A = General anesthesia, NIPPV= Noninvasive positive pressure ventilation, ICU= Intensive care unit.

54.5% (n = 48) of the patients had no postoperative complication (≥ grade II), 35.2% (n = 31) had one, 8.0% (n = 7) had two and only 2.3% (n = 2) developed three complications. The calculation of highest degree of complication showed that 39.8% (n = 35) developed a grade II complication, 3.4% (n = 3) a grade III complication and 2.3% (n = 2) grade IV as the most severe complication. A Clavien-Dindo grade III or higher (severe complications), was 5.7%. Median length of hospital stay (LOS) was 6 days (range 4–106). 85% of the patients had a LOS of 4–8 days.

### 3.3 Comparative analyses

The difference in overall complication rate (≥ grade II) between patients <60 and ≥ 60 years of age was not statistically significant. The difference in prevalence of severe complications (≥grade III) was not statistically significant either (Table 3).

**Table 3:** Comparative analyses of overall complication rate (≥ grade II) and rate of severe complications (≥ grade III) between patients <60 and ≥ 60 years of age.

Comparison		Number of patients (%)	p-value
One or more complications	<60 (n = 37)	16 (43.2)	0.89*
	≥60 (n = 51)	24 (47.1)	
Severe complication (≥ grade III)	<60 (n = 37)	3 (8.1)	0.646**
	≥60 (n = 51)	2 (3.9)	

*Chi-squared test, **Fisher's exact test.

## 4 Discussion

In this study we have demonstrated that the CDC data is feasible to retrospectively collect in a public hospital in Sri Lanka also from handwritten medical records. Our findings suggests that CDC is an approachable way to evaluate surgical quality in LMICs. This is a highly relevant finding since it is important to not only provide access, but also ensure high quality of surgical care by measurement of quality as a basis for quality improvement initiatives [4]. Previous studies on CDC have mostly been performed in high-resource settings, therefore we based on our work argue that tracking of postoperative complications using CDC should be performed also in low-resource settings as a metric to evaluate surgical quality.

After case mix and procedure stratification CDC can be used to compare postoperative outcomes in different settings. Hence, these results can be contrasted to those of *Nakanishi Et Al* [18] who investigated the influence of sarcopenia on complications after CRC surgery. In that study the prevalence of one or more complications (≥grade II) was 33% in total, compared to 45.5% in our study. Also, they found 10% ≥ grade III compared to 5.7% in this material. Though, possible sources of error are under reporting, small sample size and different case mix.

In order to make improvements, hospitals in Low- and Middle-Income Countries (LMICs) need access to tools for comparing the quality of the surgery performed. The CDC is such a tool, enabling comparisons of postoperative outcomes between different groups in the same setting as well as between settings. In this study, we observed no statistically significant difference in the rate and severity of complications between patients <60 years and ≥60 years of age. The relatively high life expectancy (74.9 years) in Sri Lanka [7] suggests that the population is fit for surgery also in higher ages. On the other hand, the growing disease burden from Non-Communicable diseases (NCDs) [7] results in additional comorbidities among the patients, which can increase the postoperative risks even in younger ages.

Furthermore, this study demonstrates that length of hospital stay (LOS) could be determined in all patients included in the study, based on retrospectively reviewed medical records. Prolonged hospital stay is associated with multiple postoperative complications and worse patient recovery [10]. Hence, these results indicates that LOS is another easily accessible indicator of surgical quality in low-resource settings.

The CDC can be used to detect complications or treatments which are overrepresented at a specific setting. In this study, the observed blood transfusion rate was as high as 22.7%. Research has shown that blood transfusions are associated with adverse outcomes, such as postoperative infections, cancer recurrence and overall mortality [[19], [20], [21]]. In order to decrease the rate of blood transfusions, it is recommended to detect and treat preoperative anemia with intravenous iron, several weeks before surgery [22]. Otherwise, there is a high risk that a preexisting anemia becomes aggravated by major surgery. As noticed during the data collection, most of the transfused patients were anemic already before surgery. Another factor which was over-represented in this study was that all incisional surgical site infections (SSIs) were treated with antibiotics, resulting in being classified as CDC grade II. However, the recommended treatment for incisional SSI is first of all debridement, without systemic antibiotics. This is important to pay attention to in the light of the growing problem with antibiotic resistance.

Our study has some limitations. Firstly, it is a relatively small cohort with inherent risk of statistical underpower. Still, we believe the results can provide an estimate of the level of occurrence of different complications and serve as a proof of feasibility. Secondly, it is single centered and retrospectively performed. Also, the coverage of the data was not complete since every event may not be documented in the paper based medical records. However, a lot of information was still possible to collect about the postoperative course, which this study well demonstrates. We believe this confirms that CDC is useable even in settings with less developed record keeping, data collection and possibilities for patient follow up. A strength with this study is that one person performed all the data collection and grading. For this reason, all patients were interpreted and evaluated in the same way. A highly relevant question is if the reporting of events is complete in this study. The overall impression is that the documentation in the medical record at Colombo South Teaching Hospital (CSTH) is detailed and comprehensive as it is an extensively used tool in the daily work by the medical staff. Therefore, we argue that the coverage of postoperative complications is adequate in this study. However, as underreporting of events is always an issue in retrospective studies a prospective study to validate these findings would be valuable.

## 5 Conclusions and implications

We believe that the present study indicates that the Clavien-Dindo Classification (CDC) can be systematically established from retrospective review of paper based medical records to measure short-term complications following colorectal cancer surgery at Colombo South Teaching Hospital (CSTH). This method of adding important data to monitor surgical quality is likely possible to reproduce in many resource-limited settings and for many conditions and procedures. The findings in this study are giving opportunities for possible quality improvement at the investigation site such as decreasing blood transfusions and minimize treatment with antibiotics. Furthermore, the results suggests that length of hospital stay (LOS) could be used as an additional metric for surgical quality in Low- and Middle-Income Countries (LMICs). The comparison of complications between patients <60 and ≥ 60 years of age is an example of how the CDC can be used to analyze subgroups. In this case, no statistically significant difference was found.

## Ethical approval

The study aligns to the principles of the Helsinki Declaration. Ethical approval was received from the Ethics Review Committee of the Colombo South Teaching Hospital (My Ref: MO/PLEC/2020). Anonymized data (patients were anonymized using unique study identification numbers).

## Sources of funding

DL is funded by the 10.13039/501100007687Swedish Medical Society and the Swedish state under the ALF agreement (ALFGBG-874451). Open access funding provided by University of Gothenburg.

## Author contribution

Stina Lindholm: Conceptualization, methodology, formal analysis, investigation, writing (original draft, review & editing), project administration. Sofia Lindskogen: Conceptualization, Methodology, Investigation, Visualization, writing (original draft, review & editing), Project administration. Göran Kurlberg and David Ljungman: Supervision, validation, writing (review & editing). Bawantha Gamage: Validation, writing (review & editing).

## Trial registry number

Name of the registry: *Clinicaltrials.gov.*

Unique Identifying number or registration ID: *NCT05182762.*

Hyperlink to your specific registration (must be publicly accessible and will be checked): https://clinicaltrials.gov/show/NCT05182762.

## Guarantor

David Ljungman and Göran Kurlberg.

## Data statement

The datasets generated and analyzed during the current study are available from the corresponding author on reasonable request.

## Provenance and peer review

Not commissioned; externally peer-reviewed.

## Declaration of competing interest

None.
